# Sparse Reconstruction for Temperature Distribution Using DTS Fiber Optic Sensors with Applications in Electrical Generator Stator Monitoring

**DOI:** 10.3390/s16091425

**Published:** 2016-09-07

**Authors:** João Paulo Bazzo, Daniel Rodrigues Pipa, Erlon Vagner da Silva, Cicero Martelli, Jean Carlos Cardozo da Silva

**Affiliations:** 1 Graduate Program in Electrical and Computer Engineering (CPGEI)/Federal University of Technology-Parana, Curitiba 80230-901, Brazil; jpbazzo@utfpr.edu.br (J.P.B.); danielpipa@utfpr.edu.br (D.R.P.); cmartelli@utfpr.edu.br (C.M.); 2 Engie Brasil Energia, Saudades do Iguaçu 85568-000, Brazil; erlon.silva@engie.com.br

**Keywords:** generator stator temperature, distributed temperature sensing, sparse reconstruction algorithm

## Abstract

This paper presents an image reconstruction method to monitor the temperature distribution of electric generator stators. The main objective is to identify insulation failures that may arise as hotspots in the structure. The method is based on temperature readings of fiber optic distributed sensors (DTS) and a sparse reconstruction algorithm. Thermal images of the structure are formed by appropriately combining atoms of a dictionary of hotspots, which was constructed by finite element simulation with a multi-physical model. Due to difficulties for reproducing insulation faults in real stator structure, experimental tests were performed using a prototype similar to the real structure. The results demonstrate the ability of the proposed method to reconstruct images of hotspots with dimensions down to 15 cm, representing a resolution gain of up to six times when compared to the DTS spatial resolution. In addition, satisfactory results were also obtained to detect hotspots with only 5 cm. The application of the proposed algorithm for thermal imaging of generator stators can contribute to the identification of insulation faults in early stages, thereby avoiding catastrophic damage to the structure.

## 1. Introduction

**Figure 1:**
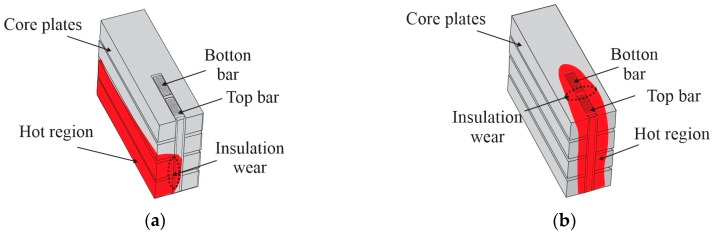
Examples of insulation faults in the generator stator. (**a**) Isolation fault between core and bars; (**b**) Isolation fault between bars.

Stator temperature is one of the most influential parameters in the degradation of hydroelectric generators [1]. High temperatures, above 100 °C, can accelerate the wear of the insulation layer of the windings, leading to premature failure and compromising the integrity of the generator [1,2]. Figure 1 shows some examples of faults that can occur due to insulation wear of the stator. Figure 1a presents a failure caused by defects in insulation between the core and the bars, which can cause partial discharges by corona effect due to potential difference between core and bars [3]. Figure 1b shows a failure caused by defects in insulation between bottom and top bars, which can also cause partial discharges due to the phase difference between the bars, increasing risk of short circuit in the stator [4,5]. In both examples, the faults cause areas of high temperature near the defect location. Thus, these faults can be identified as hotspots in certain parts of the structure. However, if the defect is not identified and repaired in the early stages, the hotspots can propagate over the structure, affecting the life expectancy of the insulation layer, leading in extreme cases to a catastrophic failure of the generator [3,4,5].

Usually, stator temperature measurements are performed through conventional localized sensors such as PT100 (Platinum Thermo-resistance) or RTD (Resistance Temperature Detector). These sensors are suitable for monitoring temperature changes during standard operation (no failure), given that the temperature distribution is considered to be rather uniform [6,7]. On the other hand, they are not able to identify hotspots that may arise in the stator coils as a result of insulation breakdown. Generally, the stator is instrumented with a few tens of localized sensors, which are not sufficient to monitor the entire structure containing hundreds of bars. Furthermore, the use of conventional electronic transducers in generators presents additional drawbacks because of their sensitivity to electromagnetic interference [6,7]. These factors motivate the use of other sensing technologies, aiming at monitoring the temperature distribution over the stator structure [8].

Recent research has shown that the distributed optical sensor technology (DTS) has great potential for applications related to monitoring of generator stator temperature [6,7,8,9,10]. DTS systems measure temperatures by means of optical fibers. These optoelectronic devices provide a continuous profile of the temperature distribution along the fiber cable [11]. Thus, the complete stator instrumentation can be carried out using only one optical fiber as a sensor, which is also immune to electromagnetic interference from the hostile environment inside the generator [11,12]. The main DTS technologies are those based on Raman and Brillouin scattering. Raman DTS systems have become popular for practical application due to their low cost and great stability when compared to equipment based on Brillouin scattering [12]. Typically, commercial Raman DTS equipment is able to provide a temperature profile along an optical fiber with over 30 km length, with accuracy of 0.1 °C and spatial resolution of 1 m [13]. The spatial resolution is defined as the spatial distance between the 10% and 90% levels of response to a temperature step. In general, for a temperature profile described by a step pulse with length smaller than the spatial resolution, the measured temperature is lower than the real temperature by a ratio of temperature step length and spatial resolution [12,13]. This parameter can be a disadvantage of Raman DTS, and sometimes limits its use in certain applications where the thermal variations occur in regions with dimensions less than 1 m. In the case of stator temperature monitoring, hotspots with dimensions in the order of centimeters are either undetected by the Raman DTS or measured incorrectly, compromising the identification of some insulation faults at an early stage.

In the last years, several studies for improvement in spatial resolution of Raman DTS systems have been presented in the literature. From the hardware viewpoint, methods based on a more efficient use of optoelectronic devices have shown significant results regarding the DTS spatial resolution (about 10 cm) [14,15,16,17]. However, such techniques often present higher costs, besides other complications that prevent their use in commercial equipment, such as increments in response time and in measurement uncertainty. Therefore, another alternative that has been investigated is the use of signal processing techniques [18,19,20,21]. These techniques have shown enhancement in DTS performance without increasing equipment costs, as this does not require physical changes in the device. Recently, the method proposed by Bazzo et al. [21], based on Total Variation deconvolution, presented great potential with regard to spatial resolution. The results showed that it is possible to measure accurately temperature variations in lengths as short as 15 cm, and to obtain significant improvements for lengths down to 5 cm.

This work proposes an image reconstruction scheme for improving the response of the thermal imaging system for generator stator using Raman DTS. The thermal images are generated by a reconstruction algorithm based on a DTS acquisition model and sparse representation theory. In this representation method, using a dictionary that contains prototype signal atoms, images are described by sparse linear combinations of these atoms. Lately, sparse representations have been successfully applied in many areas of image processing, such as denoising, inpainting and super-resolution [22,23]. To monitor the temperature distribution of the stator, we employ a sparse representation because the system can be considered a large structure at a uniform temperature, with occasional hotspots spread out in case of insulation failure (see Figure 1). The DTS readings can be viewed as degraded observation (blurred and downsampled) of the temperature distribution on stator surface. This distribution is assumed to have a sparse representation with respect to a dictionary of hotspots, which is built based on physical properties of the structure [23]. The principle of the image reconstruction ensures that under mild conditions, the sparse representation can be correctly recovered from the degraded observation (sensor readings) [23]. Due to the difficulty for reproducing insulation faults in real stator structure, the experimental tests were performed using a prototype to generate the hotspots in a similar structure. The proposed technique permits a more precise monitoring of the stator temperature distribution, facilitating the identification of insulation faults at an early stage, and preventing further damage to the generator.

This paper is organized into seven sections: Section 2 presents an overview of a thermal imaging system for generator stator, with details of real stator structure and stator prototype used in experimental tests. The details of DTS acquisition model are presented in Section 3. The dictionary of hotspots used to generate thermal images is presented in Section 4. The details of the image reconstruction algorithm are presented in Section 5, and the results are shown in Section 6. Finally, Section 7 presents the main conclusions on the results obtained.

## 2. Overview of Thermal Imaging System

**Figure 2:**
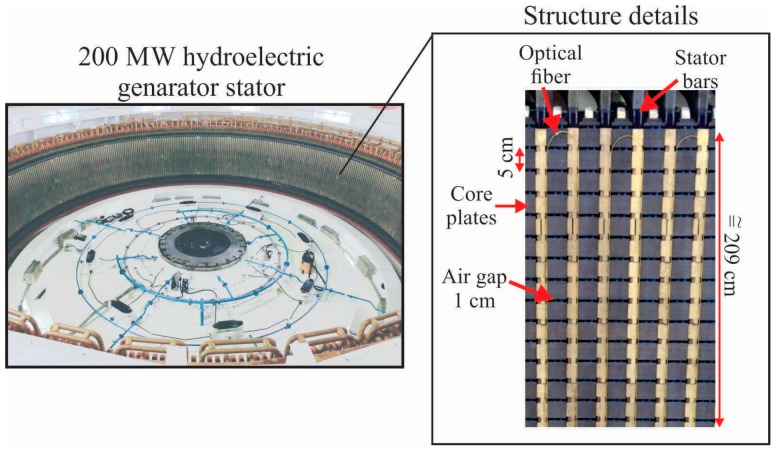
Real structure of the 200 MW hydroelectric generator stator, and fiber optic installation for the thermal imaging system using DTS.

In a previous work [6], Bazzo et al. presented a thermal imaging system for stator using DTS that was tested in a 200 MW hydroelectric generator. The main details of the structure and DTS installation on the stator surface are shown in Figure 2. As can be seen, the structure is basically composed of stacked 5 cm high bars with air gaps of 1 cm through which cooling air flow generated by the rotor circulates. The winding bars are installed in vertical slots spaced by approximately 10 cm. A distributed sensor based on fiber optics (DTS) was positioned on each slot that accommodates the winding bars, as the bars are the main source of heat structure [24,25]. Although this system has shown satisfactory results, the limitations of DTS spatial resolution impede the identification of hotspots with dimensions of less than 1 m. A similar work presented by Hudson et al. [7] also reports the need for a DTS equipment with spatial resolution about 10 cm for a more accurate thermal mapping of a generator stator. This motivates the development of an image reconstruction algorithm to improve system response, and to enable the identification of hotspots with dimensions at the order of centimeters.

**Figure 3:**
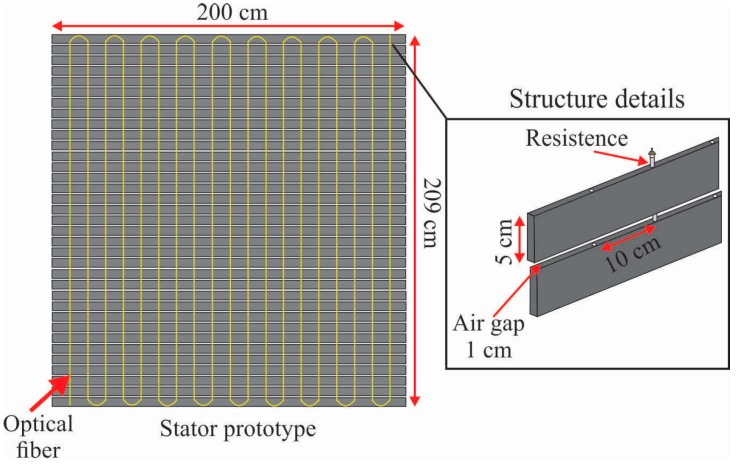
Stator prototype developed to simulate insulation faults and evaluate the imaging reconstruction algorithm performance.

To evaluate the performance of the proposed method, we developed a prototype with similar structure of the stator surface, as shown in Figure 3. The tests on the prototype were necessary since it is not possible to generate insulation faults in the generator stator. Moreover, it is noteworthy that the generator was in perfect condition and in full operation at the power plant. The stator prototype was assembled with 35 aluminum plates with dimensions 200 × 5 × 1.5 cm, stacked with air gap of 1 cm, similar to the stator core plates (Figure 2). Each plate has holes spaced at 10 cm similarly to the stator slots, and the stator bars were represented by resistances that can be embedded into the holes. Resistances with different lengths were used, and these were driven by a Proportional Integral Derivative (PID) controller. Thus, it was possible to simulate hotspots with dimensions 5 to 209 cm, in a similar structure of stator surface. The optical fiber used as distributed sensor was installed the same way as the real stator structure. As the fiber must be positioned on the main heat sources of the structure, which must be previously known, the maximum lateral displacement between the fiber loops should be 10 cm, which is a critical system parameter to ensure the resolution of thermal images. Although this structure is simple compared with the real stator structure, it reproduces the contact surface and the position where the sensor was installed in the real generator stator. Tests in the laboratory also allowed the use of a thermal camera Fluke^®^ Ti25 (Fluke Corporation, Everett, MA, USA) as a comparison reference to the thermal images generated by the image reconstruction algorithm. In the real stator structure, this would not be possible, as there is not enough space to install a camera after the rotor engagement.

The proposed image reconstruction algorithm was based on a DTS acquisition model and a dictionary of hotspots to generate thermal images. Section 3 presents more details about sensor model development.

## 3. Distributed Temperature Sensing (DTS) Acquisition Model

In a previous work [21], we showed that a linear model is suitable to represent the DTS response if one aims to reconstruct hot steps. In this work, we employ the same acquisition model, which was obtained by linear system identification techniques [26]. The input f(z) is the real temperature profile and the output g(z) is the DTS temperature readings. As we are considering the steady state, i.e., no time variations, the only independent variable is z which represents the distance (cm) along the optical fiber sensor. The response g(z) is obtained by the convolution of the DTS impulse response h(z) with input f(z), as shown in Equation (1) [26]:

(1) g(z)=h(z)∗f(z),

by applying the Laplace transform we obtain Equation (2):

(2) G(s)=H(s)F(s).

The system identification consists in estimating the poles and zeros of a transfer function H(s), as shown in Equation (3) [26]:

(3) $$H\left(s\right)=\frac{b_{0}s^{m}+b_{1}s^{m-1}+\;\ldots +b_{m}}{s^{n}+a_{1}s^{n-1}+\;\ldots +a_{n}}=\frac{\prod_{i=1}^{m}\left(s-\beta_{i}\right)}{\prod_{i=1}^{n}\left(s-\alpha_{i}\right)},$$

where *β_i_* are the zeros and *α_i_* are the poles.

**Figure 4:**
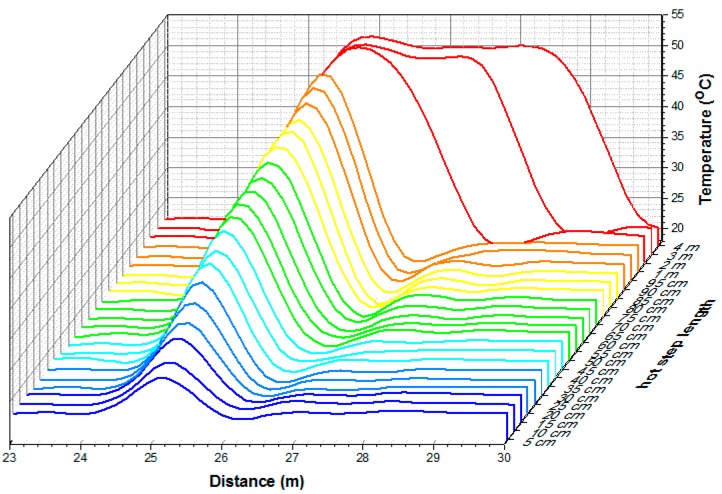
Experimental results used to identify the DTS system model, where the hotspots were at 50 °C with lengths from 5 cm to 4 m, with intervals of 5 cm.

The DTS equipment used in this work was an AP Sensing^®^ N4385B (AP Sensing GmbH, Böblingen, Germany) model. This model features a spatial resolution of 1 m, acquisition time of 30 s, sample interval down to 15 cm, and temperature resolution of 0.04 °C for fibers of up to 2 km. To evaluate the equipment response, an experimental test was carried out in a thermal bath LAUDA^®^ECO RE415G model, with stabilized temperature at 50 °C, providing hotspots of different lengths. The ambient temperature was 21.7 °C. The input f(z) and output g(z) were obtained for hotspots at 50 °C with lengths from 5 cm to 4 m with intervals of 5 cm, as shown in Figure 4. As can be seen, for hotspots of 5 cm the measured temperature was only 24.7 °C. From 1 m and above, the temperature is measured correctly (50 °C), confirming the spatial resolution specification of the DTS equipment.

**Figure 5:**
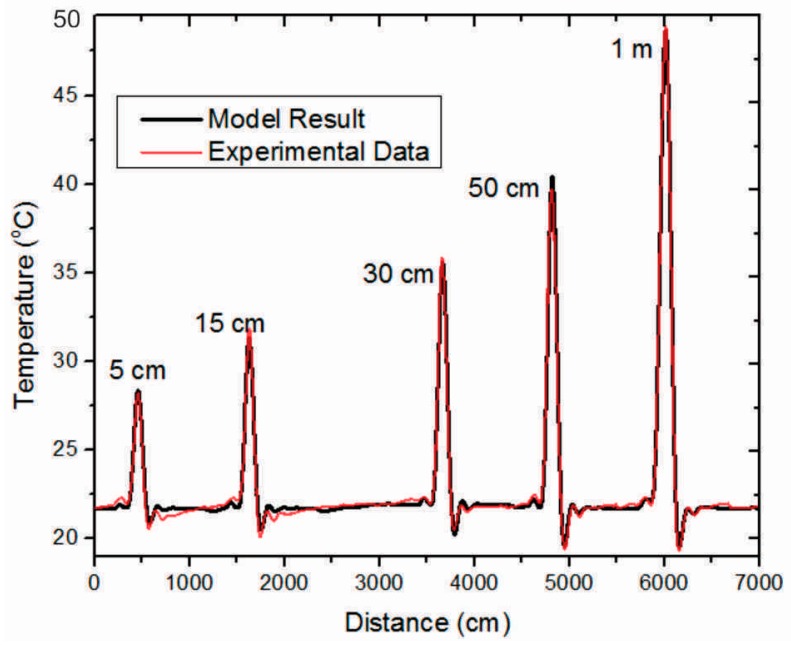
Experimental results vs. DTS acquisition model.

We employed the prediction error minimization (PEM) approach to estimate the transfer function coefficients. In this case, from initial estimates, the parameters are updated using a nonlinear least-squares search method, where the objective is to minimize the weighted prediction error norm [26]. As a result, we obtained a transfer function with nine poles and four zeros (determined empirically) and 98% accuracy. The comparison between experimental data and model simulation is shown in Figure 5.

**Figure 6:**
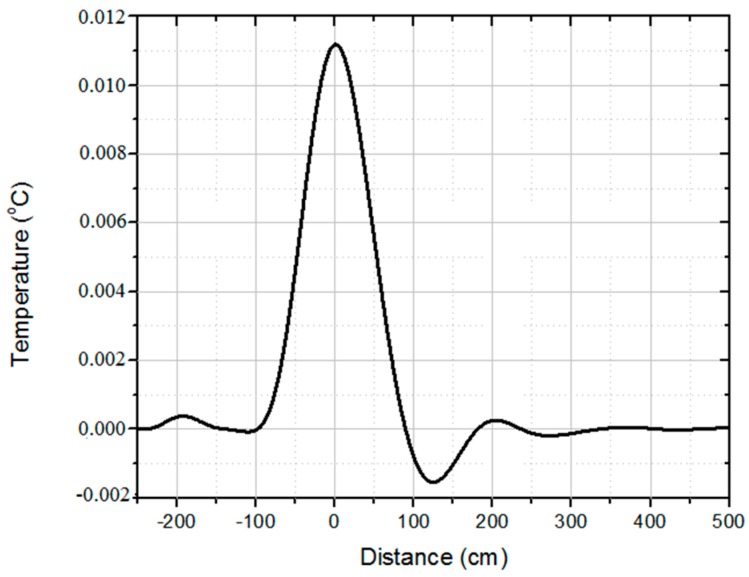
Experimental results vs. DTS acquisition model.

Taking the inverse Laplace transform of the transfer function, we get the impulse response of the system h(z), presented in Figure 6. The DTS impulse response h(z) is used to assemble a sensitivity matrix **H**, which represents the DTS acquisition model. The matrix-vector notation of **H** is presented in Equation (4):

(4) $$H=\left[\begin{array}{c} h\left(z_{0}\right)\\ \vdots \\ \vdots \\ h\left(z_{k}\right) \end{array}\begin{array}{c} h\left(z_{0-1}\right)\\ h\left(z_{0}\right)\\ \vdots \\ h\left(z_{k-1}\right) \end{array}\begin{array}{c} \cdots \\ \cdots \\ \ddots \\ \cdots \end{array}\begin{array}{c} h\left(z_{0-k}\right)\\ \vdots \\ \vdots \\ h\left(z_{0}\right) \end{array}\right].$$

**Figure 7:**
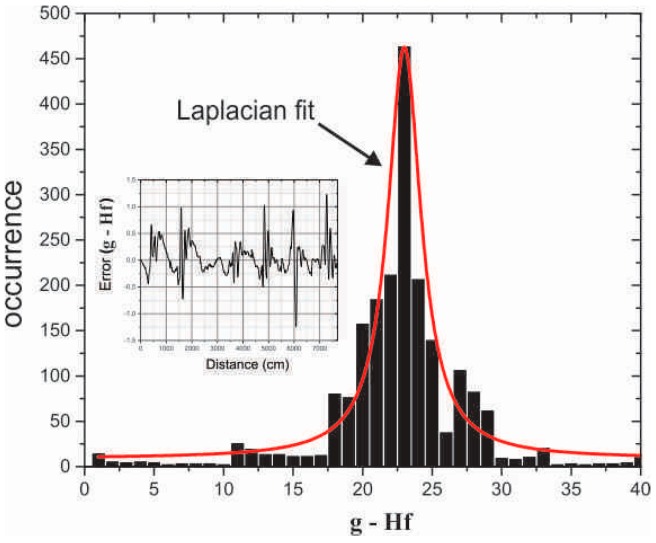
Histogram of the residuals for the DTS model and a Laplacian curve fit.

Although the matrix **H** has proven suitable for representing the sensor acquisition, the DTS model contains errors and is itself a source of noise, as can be seen in Figure 5. To develop an efficient reconstruction algorithm, a statistical analysis of the noise is fundamental to set the norm in the data term of the cost function [22,27]. Thus, an analysis of the residuals was performed by the histogram **g**-**Hf**, where **g** is a vector formed by the sensor data (DTS readings), and **f** is a vector representing the temperature profile. Figure 7 summarizes the analysis results. Although the histogram presents a slight skew toward high residual values, thereby indicating large model errors, this misfit is relatively rare (see bar height). This behavior is expected when adopting linear models (for tractability purposes) where the underlying physics is potentially nonlinear. To accommodate this inaccuracy, we performed the following statistical analysis, similarly to [28]. Assuming a generalized Gaussian distribution, we obtained a shape parameter p≈1 using the method described in [27], indicating that the residuals have a Laplacian distribution. This information will be further exploited in Section 5.

## 4. Dictionary of Hotspots

**Figure 8:**
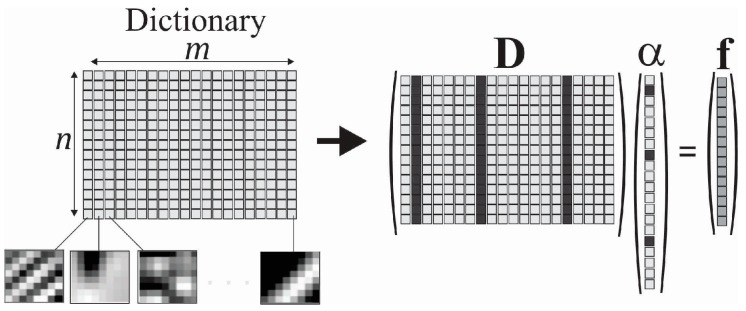
Example of the sparse representation of an imaging system using a dictionary.

We assume that the thermal system for generator stator can be modeled through a sparse representation. The reason is that it can be considered a large structure with uniform distribution temperature, where occasional hotspots may arise in case of insulation failure (Figure 1). Using a dictionary matrix $D\in {\mathbb{R}}^{m\times n}$ that contains *n* prototype image atoms in the columns ${\{d_{i}\}}_{j=1}^{n}$, a thermal image $f\in {\mathbb{R}}^{m}$ can be represented by sparse linear combinations of the dictionary atoms, as shown in Equation (5) [29]:

(5) f=Dα,

where the vector $\alpha \in {\mathbb{R}}^{n}$ contains the coefficients. Each atom is the thermal image of the whole structure when only one possible heat source is active at a time. In this representation **α** is sparse, i.e., it is assumed to contain mostly zeros. The representation of **f** may either be exact **f** = **Dα** or approximate f≈ **Dα**, satisfying Equation (6) [29]:

(6) $$∥f-D\alpha {∥}_{p}\;\leq \epsilon ,$$

where ϵ is the minimum residual error desired, and p is the norm used for measuring the deviation. Figure 8 shows an example of the sparse representation of an imaging system using a dictionary. In this example, a combination of three atoms of the dictionary **D** was used to form the image **f** [29,30].

We built a dictionary of hotspots through simulation using the COMSOL^®^ (Comsol, Stockholm, Sweden) multiphysics tool. In the simulations, we considered the physical properties of the materials, geometry and environment boundary conditions. Each atom was generated considering the position of the resistances in each plate. As the prototype has 35 plates at 5 cm high, and 19 positions for resistance, considering 1 atom for each 1 cm, we generated 3325 atoms (35 × 5 × 19) to model the system. Although this representation of 1 cm results in a large number of atoms, it was necessary to reconstruct hotspots with more accurate dimensions, also preventing alignment problems of the sensor installation. Since the total size of the image 209 × 200 cm, the vector length of each atom is 41,800, forming a dictionary matrix **D** of 41,800 × 3325.

**Figure 9:**
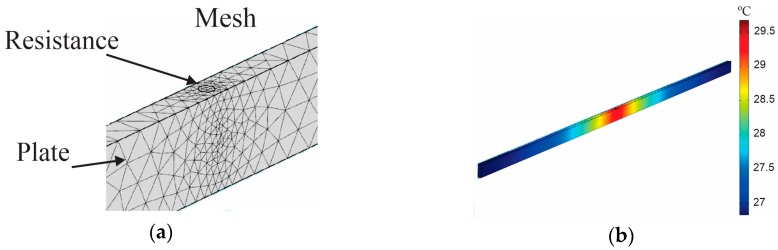
Multiphysics simulation of stator prototype. (**a**) Mesh geometry used in the simulations. (**b**) Temperature distribution for total power of 1 W/cm^3^ applied to resistance at 26 °C ambient temperature.

The columns of the dictionary matrix, or atoms, were formed by the temperature distribution values generated by power of 1 W/cm^3^ applied to a resistance of 5 cm in each position along the plates, considering steady state. We set the resistance of 5 cm as a minimum condition, because of the plate dimensions and difficulty in using tubular resistances with lower length. However, the temperature data is sampled every 1 cm for forming atoms, as explained in the previous paragraph. Thus, the vector **α** represents the values of the thermal power that generate the hotspots in the structure. Therefore, by estimating **α,** we obtain the location and the amount of thermal power that causes each hotspot. Figure 9a shows details of the mesh geometry used in the simulations, and Figure 9b shows the temperature distribution for total power of 1 W/cm^3^ at 26 °C ambient temperature, in steady state. The results show a variation of 2.62 °C with decreasing temperature of $e^{-x/45}$, where x is the distance from the heat source. This decreasing function is a parametric fit of the numerical simulations, which was used to ease the construction of the dictionary. The application of the dictionary **D** in the image reconstruction algorithm is discussed in more detail in Section 5.

## 5. Imaging Reconstruction Algorithm

First, considering the basic model of image reconstruction theory, the acquisition system can be represented by Equation (7) [22]:

(7) g=Hf+n,

where g is a vector formed by DTS readings, H is the sensitivity matrix, f is a vector representing the temperature distribution in stator surface, and **n** is a vector representing all sources of additive noise. Considering that the thermal image has a sparse representation in the constructed dictionary, the acquisition system can be rewritten substituting Equation (5) into Equation (7), as shown in Equation (8) [22]:

(8) g=HDα+n.

As shown in Figure 6, the DTS “spreads” the impulse, which is a characteristic of low-pass systems. This is translated into an ill-conditioning of matrix **H**. Thus, the recovering of the temperature distribution by simple inversion of Equation (8) yields high noise amplification, generating poor results [21]. This problem requires regularization, which stabilizes the reconstruction and improves the results. Although the dictionary has been built with the hotspots of 1 cm to improve representation, we expected them to occur with 5 cm or more (see Figure 9), because of the minimal condition of structure prototype and steady state, as explained in Section 4. Therefore, the most appropriate regularization is Total Variation, as it privileges piece-wise constant signals. Thus, the cost function to solve the inverse problem is given by Equation (9) [21,22]:

(9) $$\hat{\alpha }=\underset{\alpha }{\mathrm{argmin}}∥g-HD\alpha {∥}_{p}^{p}+\lambda ∥Q\alpha {∥}_{1},$$

where $\hat{\alpha }$ is a vector with the values of the thermal power that generate the hotspots in the structure, p is the norm used in the data-fidelity term, λ is the regularization parameter which controls the sensitivity of the solution to the noise, and **Q** is a finite difference matrix. In approximation methods, typical norms used for measuring the deviation are the $L_{p}$-norms for p = 1, 2 and ∞. It is common to use the L_2_ norm in the data term because the noise is usually well represented by a normal distribution [27,29]. However, according to the statistical analysis presented in Section 3, the DTS acquisition model contains residual errors with Laplacian behavior, which indicates that the L_2_ norm should be replaced by an L_1_ norm, i.e., *p* = 1 [27]. To solve Equation (9) with *p* = 1, we used the Interactive Reweighted Least Squares (IRLS) approach. This method consists of approximating the cost function by weighted quadratic L_2_ norms, updating the solution by solving a least squares problem and reiterating those two steps until some stop criterion is attained, usually defined by a minimum update rate [31]. The implementation details in Matlab^®^ (R2014a, MathWorks, Natick, MA, USA) are shown in Algorithm 1.

**Algorithm 1:** Image Reconstruction Algorithm
**Require:**
**g**% DTS readings, **H**% sensitivity matrix, **D**% Dictionary of hotspots, **Q**% finite difference matrix**Require:** λ % Regulariztion parameter (set empirically)**Require:** e=10^–9^ % avoids zero division**Require:**
**H_D_** = **H** × **D** % impulse response of the dictionary**Require:**
$\hat{\alpha }$ = **H_D_′** × **g** % intial solution**Require:**
ϵ % minimum update to stop
1:   **while** stop > ϵ
2:    ${\hat{\alpha }}_{0}$**=**$\hat{\alpha }$3:    **Wh** = diag(1 ./ (abs(**g** − **H_D_** × $\hat{\alpha }$) + e)) % data term weights4:    **Wl** = diag(1 ./ (abs(**Q** × $\hat{\alpha }$) + e)) % penalization term weights5:    $\hat{\alpha }$= (**H_D_′** × **Wh** × **H_D_** + λ × (**Q′** × **Wl** × **Q**))\(**H_D_′** × **Wh** × **g**) % least-squares6:    stop = norm($\hat{\alpha }$ − ${\hat{\alpha }}_{0}$)/norm(${\hat{\alpha }}_{0}$) % stopping criterion
7:   **end while**
8:  **f** = reshape(**D** × $\hat{\alpha }$,209,200) % thermal image

The proposed image reconstruction algorithm was evaluated with simulated data to assess the robustness to different noise levels, and with experimental data through the stator prototype (Figure 3). The results are shown in Section 6.

## 6. Results

This section is organized into two subsections: Subsection 6.1 presents the results obtained simulating the response **g** by sensitivity matrix **H** for a given hotspot Dα, in order to assess the algorithm robustness applying different noise levels; Subsection 6.2 shows the results obtained by the experimental tests with the stator prototype, using resistances to emulate heat sources of different lengths.

### 6.1. Simulated Results

**Figure 10:**
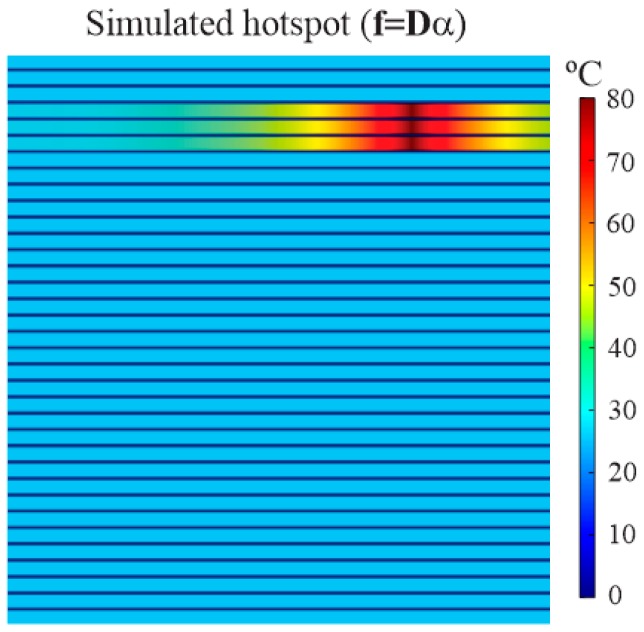
Simulated hotspot image on three plates, where the ambient temperature was 26 °C, the maximum temperature was 80 °C and the length approximately 15 cm.

The evaluation of the algorithm performance with respect to noise level was conducted simulating a hotspot with the dictionary D. The simulated hotspot covered a region of three plates, with a maximum temperature of 80 °C and ambient temperature of 26 °C, which represents a length of approximately 15 cm, considering the fiber installation (Figure 3). This length was chosen based on spatial resolution achieved with the Total Variation deconvolution method proposed in [21]. The simulated hotspot image **f** is shown in Figure 10.

Based on the image of Figure 10, the response **g** was obtained using the sensitivity matrix **H** of the DTS acquisition model presented in Section 3. Thus, the algorithm performance was evaluated by adding white Gaussian noise at different levels to the simulated DTS readings. The images reconstructed for each noise level were compared with the ground-truth hotspot images. We employed the mean square error (MSE) and the maximum temperature difference (MTD) as figures of merit.

**Figure 11:**
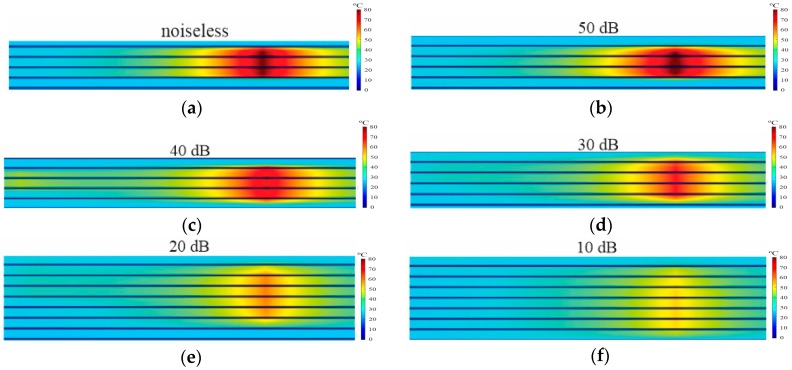
Simulated hotspot image on three plates, where the ambient temperature was 26 °C, the maximum temperature was 80 °C and the length approximately 15 cm. (**a**) Reconstructed hotspot without adding noise; (**b**) Reconstructed hotspot with SNR 50 dB; (**c**) Reconstructed hotspot with SNR 40 dB; (**d**) Reconstructed hotspot with SNR 30 dB; (**e**) Reconstructed hotspot with SNR 20 dB; (**f**) Reconstructed hotspot with SNR 10 dB.

The reconstructed images are shown in Figure 11, and a brief analysis of results is presented in Table 1. In the first test, Figure 11a, no noise was added to the simulated DTS readings, and the reconstructed image presented reduced errors relative to simulated hotspot, with only 0.1 °C uncertainty. In tests adding a certain noise level (Figure 11b–f), it can be seen that is possible to obtain, from data with SNR up to 40 dB, acceptable images for application in the stator, with less than 4 °C uncertainty for hotspots of 15 cm. However, from data with SNR between 30 dB and 10 dB (Figure 11d–f), besides large errors (up to −23.7 °C) in the temperature estimation, other parameters such as location and dimension become affected. As can be observed, the reconstructed hotspot covered four plates instead of threee plates of the original hotspot.

Another numerical analysis was performed to assess the minimum length of a detectable hotspot given a maximum acceptable temperature difference of ±1 °C and different SNR levels. Table 2 summarizes the results, where it can be seen that the minimum length for the given conditions was 15 cm with SNR 50 dB. For other SNR levels, 40 dB and 30 dB, the performance was affected and the minimum length increased to 22 cm and 27 cm, respectively. Subsection 6.2 presents the results of the experimental tests’ performance using the stator prototype.

### 6.2. Experimental Results

The experimental results were obtained by tests with the stator prototype (Figure 3) and a thermal camera Fluke^®^ Ti25 (Fluke Corporation, Everett, MA, USA), which was used as reference for the reconstructed images. In addition to the comparison with the thermal camera, the images generated by the sparse reconstruction algorithm were also compared with the linear interpolation method used in the thermal imaging system presented in [6]. Three resistances with different lengths were used as heat sources, which produced hotspots with approximately 100 cm, 15 cm and 5 cm.

**Figure 12:**
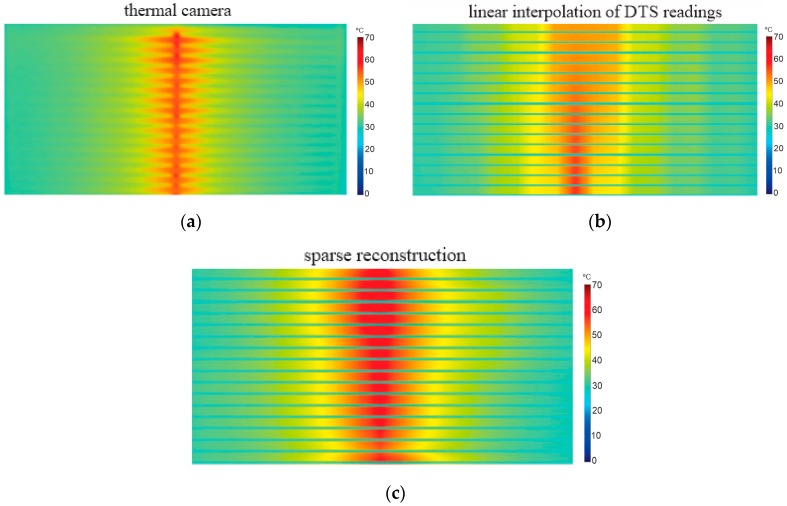
Experimental results for hotspot of 100 cm, with maximum temperature of 63.7 °C and ambient temperature of 27.8 °C. (**a**) Reference image taken by thermal camera; (**b**) Image generated by the linear interpolation method using raw DTS readings [6]; (**c**) Image reconstructed by proposed algorithm.

The test results for a hotspot of 100 cm is shown in Figure 12. In this test, the ambient temperature was 27.8 °C; the total power applied to the resistance was 680 W (≅12 W/cm^3^), generating a hotspot with maximum temperature of 63.7 °C after entering in steady state. The image taken by the thermal camera is presented by Figure 12a, where the temperature distribution in 17 plates of the stator prototype is observed. Figure 12b shows the image generated by linear interpolation of raw DTS readings, where it can be seen that the maximum temperature was measured in an approximate way for only seven plates (MTD of *−*2.8 °C), while for the rest of the plates, the MTD was up to *−*14 °C. This is because the unprocessed DTS readings are strongly influenced by spatial resolution (Figure 4), generating a blurred image. The image reconstructed by the proposed algorithm is represented in Figure 12c. In this result, the maximum temperature was 63.2 °C (MTD of *−*0.5 °C), and both length and location of the hotspot were in accordance with the image of thermal camera. As can be seen, the sparse reconstruction showed significant improvements when compared to the linear interpolation method, even for a hotspot with dimensions in line with the DTS spatial resolution (1 m). Although there are some differences in the temperature distribution along the plates, the main parameters of interest for application on stator structure are quite accurate, namely the location, length and maximum temperature.

**Figure 13:**
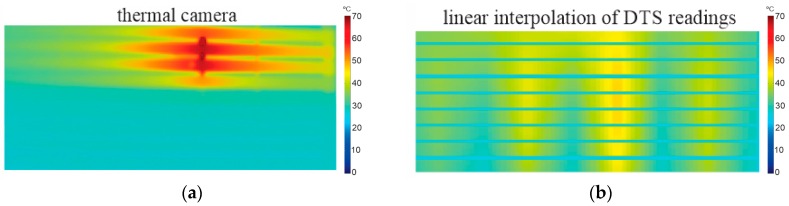

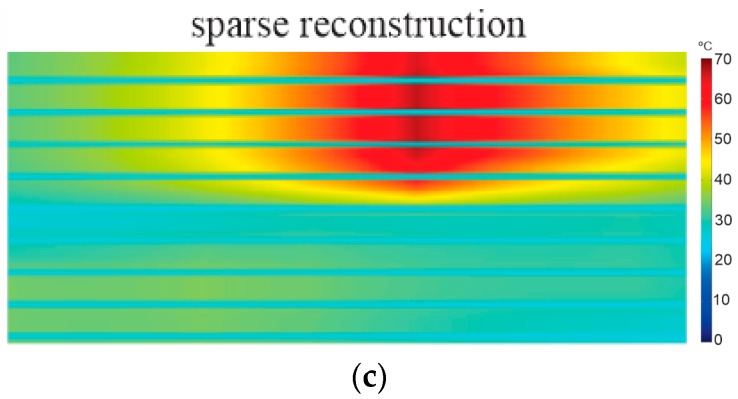
Experimental results for hotspot of 15 cm, with maximum temperature of 67.5 °C and ambient temperature of 25.9 °C. (**a**) Reference image taken by thermal camera; (**b**) Image generated by the linear interpolation method using raw DTS readings [6]; (**c**) Image reconstructed by proposed algorithm.

Another test was conducted using a hotspot with dimensions smaller than the DTS spatial resolution. In this experiment, we applied a total power of 120 W (≅15 W/cm^3^) at a resistance of 15 cm, for which the minimal length estimated in the tests is presented in Section 6.1. The generated hotspot was of 67.5 °C and an ambient temperature of 25.9 °C, as shown in Figure 13a. Figure 13b shows the image generated by the linear interpolation method, where the maximum temperature measured was only 46.1 °C (MTD *−*21.4 °C), and both the dimensions and the location of the hotspot were not correctly identified. This poor result is expected because this method uses the raw sensor readings and the hotspot length was only 15 cm, i.e., considerably lower than the spatial resolution of 1 m. The image generated by the proposed reconstruction algorithm is represented in Figure 13c. In this result, the maximum temperature was 66.8 °C (MTD of *−*0.7 °C), and location of the hotspot was in accordance with the image of the thermal camera. The length measured was about 22 cm, which can be considered a small difference, since that the dimension of the hotspot was six times smaller than DTS spatial resolution. Regarding the application in the generator stator, the proposed algorithm provides a great improvement when compared with the linear interpolation method, because the insulation faults can occur in just a few core plates (Figure 1), so it is important to perform reliable measurements for hotspots with dimensions less than 1 m.

**Figure 14:**
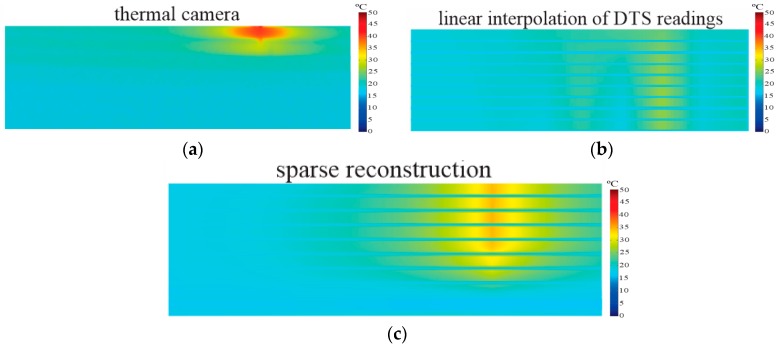
Experimental results for a hotspot of 5 cm, with maximum temperature of 50.6 °C. (**a**) Reference image taken by thermal camera; (**b**) Image generated by the linear interpolation method using raw DTS readings [6]; (**c**) Image reconstructed by proposed algorithm.

A test to evaluate an extreme case for the image reconstruction algorithm was performed producing a hotspot of only 5 cm. In this test we applied a total power of 25 W (≅9 W/cm^3^) on a resistance positioned in one of the plates, which generated a hotspot with maximum temperature of 50.6 °C and an ambient temperature of 22.8 °C. The thermal image taken by the thermal camera is presented in Figure 14a. The result of the linear interpolation method is shown in Figure 14b. As can be seen, the generated image is extremely blurred and it was not possible to identify the hotspot. This result shows that the use of Raman DTS becomes impractical for measurements in the order of 5 cm without using signal reconstruction techniques. The image generated by the reconstruction algorithm is represented in Figure 14c. In this result, the maximum temperature was 41.5 °C (MTD of −9.1 °C). Besides the large difference in temperature, the hotspot length was also measured incorrectly, which was 25 cm instead of 5 cm. As can be seen, the hotspot is spread on five plates when it should be only on one plate. This is mainly due to the DTS spatial resolution that affects the signal-to-noise ratio (SNR), spreading and attenuating the sensor response, as already shown in Subsection 6.1, more specifically in Figure 11e,f. Although in this case the algorithm does not provide precise measurements of temperature and dimension, it is possible to identify the existence and approximate location of a fault, even for hotspots with dimensions up to 20 times smaller than the DTS spatial resolution. Section 7 presents the main conclusions on the proposed reconstruction method for thermal imaging of generator stators.

## 7. Conclusions

This paper presented an image reconstruction method that can be a promising solution for thermal imaging systems based in Raman distributed temperature sensing (DTS). The reconstruction was based on sparse representations, which has proved suitable for the application. The main advantage is the possibility to correctly identify heat sources and hotspots smaller than the DTS spatial resolution (1 m). To reconstruct the thermal images, we employed a dictionary of hotspots built from a multiphysical model of the monitored structure. Tests were performed using a prototype with structure similar to surface of a 200 MW hydroelectric generator stator. This facilitated the laboratory tests and allowed the comparison between reconstruction images and images from a thermal camera used as reference.

The evaluation of the algorithm performance with respect to noise level was conducted through simulations with the DTS model and dictionary of hotspots. These simulations shown that when signal-to-noise ratio (SNR) is up to 40 dB it is possible to obtain acceptable images for application in the stator, with less than 4 °C uncertainty. However, with SNR between 30 dB and 10 dB besides the uncertainty in the temperature measurement, other parameters such as location and dimension become affected. This provides a lower bound to which the proposed method is applicable.

The experimental results were achieved by generating hotspots of different dimensions in the stator prototype. These results show that it is possible to identify hotspots with dimensions as short as 15 cm, with temperature uncertainty of less than ±1 °C, which represents a great advance considering the DTS spatial resolution. It was also observed significant improvements for hotspot down to 5 cm. In this critical case, despite a maximum temperature difference of almost −10 °C, it was possible to identify the existence and approximate location of hotspots with dimensions up to 20 times smaller than the DTS spatial resolution.

Regarding the application for imaging system for generator stators, improvements in image resolution can help identify wear on the insulation layer in early stages, facilitating maintenance and avoiding further damage to the structure, such as short circuit in the stator windings. The accurate temperature monitoring of the stator structure can be a fundamental tool of predictive maintenance, to ensure the performance and operational availability of the generator.

## Figures and Tables

**Table 1:** Algorithm performance with respect to the noise level.

Noise Level (SNR)	MSE (mean square error)	MTD (Maximum Temperature Difference)
Noiseless	0.5657	0.1 °C
50 dB	2.0921	−0.5 °C
40 dB	6.9929	−3.6 °C
30 dB	9.3758	−12.1 °C
20 dB	16.595	−20.9 °C
10 dB	21.8149	−23.7 °C

**Table 2:** Algorithm performance with respect to the hotspot length and SNR level.

SNR	Hotspot Length	MTD (Maximum Temperature Difference)
50 dB	15 cm	−0.5 °C
50 dB	14 cm	−3.7 °C
50 dB	13 cm	−5.1 °C
40 dB	22 cm	−0.9 °C
40 dB	21 cm	−1.6 °C
40 dB	20 cm	−2.4 °C
30 dB	27 cm	−0.7 °C
30 dB	26 cm	−1.8 °C
30 dB	25 cm	−2.6 °C
